# Identification of a novel ER-NFĸB-driven stem-like cell population associated with relapse of ER+ breast tumors

**DOI:** 10.1186/s13058-022-01585-1

**Published:** 2022-12-08

**Authors:** Svetlana E. Semina, Luis H. Alejo, Shivani Chopra, Nidhi S. Kansara, Irida Kastrati, Carol A. Sartorius, Jonna Frasor

**Affiliations:** 1 Department of Physiology and Biophysics, College of Medicine, University of Illinois at Chicago, 909 S Wolcott Avenue (MC 901), 2040 COMRB, Chicago, IL 60612 USA; 2 Present Address: Department of Cancer Biology, Loyola University Chicago, Maywood, IL 60153 USA; 3 Department of Pathology, University of Colorado Anschutz Medical Campus, Aurora, CO 80045 USA

**Keywords:** Breast cancer, Estrogen receptor, Stem cells, NFĸB, Single-cell RNA sequencing

## Abstract

**Background:**

Up to 40% of patients with estrogen receptor-positive (ER+) breast cancer experience relapse. This can be attributed to breast cancer stem cells (BCSCs), which are known to be involved in therapy resistance, relapse, and metastasis. Therefore, there is an urgent need to identify genes/pathways that drive stem-like cell properties in ER+ breast tumors.

**Methods:**

Using single-cell RNA sequencing and various bioinformatics approaches, we identified a unique stem-like population and established its clinical relevance. With follow-up studies, we validated our bioinformatics findings and confirmed the role of ER and NFĸB in the promotion of stem-like properties in breast cancer cell lines and patient-derived models.

**Results:**

We identified a novel quiescent stem-like cell population that is driven by ER and NFĸB in multiple ER+ breast cancer models. Moreover, we found that a gene signature derived from this stem-like population is expressed in primary ER+ breast tumors, endocrine therapy-resistant and metastatic cell populations and predictive of poor patient outcome.

**Conclusions:**

These findings indicate a novel role for ER and NFĸB crosstalk in BCSCs biology and understanding the mechanism by which these pathways promote stem properties can be exploited to improve outcomes for ER+ breast cancer patients at risk of relapse.

**Supplementary Information:**

The online version contains supplementary material available at 10.1186/s13058-022-01585-1.

## Background

Breast cancer is the second leading cause of cancer-related mortalities in women of the US [1]. Approximately 75% of breast cancers express estrogen receptor α (ER) [2] and rely on estrogens for their proliferation, survival, and progression; therefore, inhibition of ER activity or estrogen production are common therapeutic strategies. Although most women initially respond to these endocrine therapies, up to 40% of ER-positive (ER+) breast cancer patients will experience relapse after 5 years of treatment [3–5]. A major cause of breast cancer relapse is thought to be the presence of a stem-like cell population (i.e., breast cancer stem cells (BCSCs)) that can escape therapy.

Characteristics of BCSCs include quiescence/slow proliferation, self-renewal, tumor-initiation, and therapy-resistance (reviewed in [6]). Two additional characteristics make the study of BCSC biology challenging—heterogeneity [7, 8] and plasticity [9–12]. For example, it was shown that BCSCs derived from different breast cancer molecular subtypes are characterized by different molecular markers [7], whereas several BCSC populations with different biological functions and transcriptional regulators within one mouse model of breast cancer have been described [8]. In addition to heterogeneity, BCSCs also display plasticity, which allows BCSCs to switch between distinct phenotypic states that lead to disease progression and metastasis. Two types of plasticity have been described for BCSCs, the transition between quiescent mesenchymal- and proliferative epithelial-like states [9–11] and the conversion of non-stem to stem cells [12]. Thus, BCSCs represent a heterogeneous and dynamic cell population rather than a stable population with a fixed phenotype.

The role of ER creates an additional layer of complexity in our understanding of the biology of BCSCs derived from ER+ breast tumors. Some studies showed that estrogen treatment increases BCSC properties, including mammosphere forming efficiency and the proportion of CD44+/CD24− cells, in ER+ breast cancer cell lines and that this effect was reversed by endocrine therapy, suggesting that ER is necessary for stem cell expansion [13, 14]. In addition to these studies, it was shown that mammospheres derived from several ER+ breast cancer patient tumors retain ER expression and activity [15]. Moreover, it was reported that ER+ breast cancer cells respond to estrogen by secreting growth factors that act as paracrine mediators to promote stem properties [13, 14, 16]. In addition to these studies, it has also been suggested that the ERα-36 variant can promote BCSC self-renewal and increase the CD44+/CD24− cell population [17, 18]. On the other hand, several studies have reported that activation of ER inhibits several stem cell-associated markers (NANOG, OCT4, SOX2, etc.) and pathways, decreases mammosphere formation, and reduces the pool of BCSCs [19, 20], suggesting that ER activation can also suppress stem cell properties. This concept is supported by several reports showing that ER antagonism with endocrine therapy leads to an enrichment of BCSCs [21] that expand with the development of endocrine therapy resistance [22]. Additionally, some studies have shown that BCSCs derived from ER+ breast tumors generally lack expression of ER [23–28], suggesting ER-independent mechanisms of BCSC promotion. Thus, further clarification on the role of ER in BCSCs is needed.

Here, we used a combinatorial approach to identify and define drivers of BCSCs in ER+ breast cancer cell lines. Using single-cell RNA sequencing (scRNA-seq), we identified a quiescent stem-like population of cells that is highly enriched in mammospheres. Applying various bioinformatic methods, we identified ER and NFĸB as key drivers of this stem-like population. Additionally, we derived a gene signature from this stem-like population and found that it is enriched in a subset of endocrine therapy-resistant cells, circulating tumor cells, and metastatic cell populations from PDX tumors. We also demonstrated that this signature is associated with aggressive disease and an increased risk of relapse in patients with ER+ breast cancer. Taken together, these findings implicate a newly identified quiescent stem-like cell population in the progression of ER+ breast tumors, and targeting this population could improve the outcome for breast cancer patients.

## Materials and methods

### Reagents

17β-estradiol (E2) was purchased from Sigma. TNFα (#210-TA) and GDF15 antibody (#AF957) were purchased from R&D Systems. ICI182,780 (ICI) (# I4409) and IKK7(#S2882) were purchased from Sigma and SelleckChem, respectively.

### Cell lines and culture conditions

The human ER+ breast cancer cell lines, MCF-7 and T47D, were obtained from Dr. Debra Tonetti (University of Illinois at Chicago) and authenticated. These cells are routinely maintained in RPMI 1640 media (Invitrogen Life Technologies) with phenol red supplemented with 10% FBS, 1% non-essential amino acids, 2 mmol/L L-glutamine, 1% penicillin–streptomycin, and 6 ηg/mL insulin. The ER+ breast cancer PDX cell lines, UCD4 and UCD65, were kindly provided by Dr. Carol Sartorius (University of Colorado Anschutz Medical Campus) [29] and grown in Dulbecco’s modified minimum essential medium (DMEM)/F12 with 10% fetal bovine serum, cholera toxin (100 ng/mL), hydrocortisone (1 μg/mL), insulin (1 nM), and 1% penicillin–streptomycin.

### Establishing dual reporter cell lines

MCF-7 and T47D dual reporter cell lines were generated by stable transfection with p3X-ĸB-RE-eGFP and pS2-ERE-mCherry reporter plasmids using Lipofectamine 2000 according to the manufacturer’s protocol. The p3X-ĸB-RE-eGFP reporter plasmid was obtained from Dr. Elaine T. Alarid (University of Wisconsin-Madison) [30]. The pS2-ERE-mCherry plasmid was constructed by cloning (ERE)_2_-pS2-CAT element from donor plasmid [31] obtained from Dr. Benita Katzenellenbogen into pmCherry-1 plasmid (Takara, # 632525) with XbaI and NheI restriction sites. After transfection cells were selected with geneticin and puromycin and cloned by limiting dilutions in 96-well plates. Representative clones were selected based on mCherry and GFP expression after treatment with E2 or TNFα, respectively. Reporter activity was calculated as the confluence for each fluorescent signal over the total confluence using a Celigo imaging cytometer. In addition to mCherry and GFP expression, MFE was measured for each clone and clones representative of bulk cell lines were chosen for the further experiments.

### RT-quantitative PCR (QPCR)

Total RNA was isolated using Trizol and RT-QPCR performed as previously described [32]. Fold change was calculated using the ΔΔCt method with 36B4 serving as the internal control. All QPCR primers were obtained from Origene.

### Mammosphere (MS) assay

Mammosphere assays were conducted as described [33]. Briefly, cells were seeded at single-cell density 400 cells per well on low attachment 96 well plates in media described by Dontu et al. [34], supplemented with 1% methyl cellulose to prevent cellular aggregation. After 7 days, the number of MS ≥ 75 μm in diameter was determined using a Celigo imaging cytometer, and MS forming efficiency (MFE) was calculated. Secondary MS assays were performed to validate MS forming properties of sorted cell populations, since it was shown [35, 36] that additional rounds of MS assays further enrich for cells with BCSC properties. Secondary MS assays were conducted by sorting of primary MS cells into 4 population based on ERE and NFĸB-RE reporter activity, each population was seeded as primary MS. After 7 days secondary MFE was calculated for each population. For EdU labeling, MS were cultivated with EdU reagent (10uM) for the last 72 h of the assay. Then MS were collected and transferred to 8 well glass chamber slides (Corning, #354114) for 4 h to attach. MS were then fixed with 4% PFA for 30 min, permeabilized with 0.2% Triton × 100 in TBS and labeled using Click-iT EdU Alexa Flour 633 imaging kit (Invitrogen, # C10337) according to manufacturer’s protocol. Cells were imaged with a Zeiss LSM880 confocal macroscope.

### Western blot

Nuclear lysates were collected using NE-PER kit (Thermo Scientific). Proteins were separated by SDS-PAGE (Bio-Rad Laboratories), transferred to nitrocellulose membranes (Thermo Scientific), blocked for 1 h in buffer containing 5% nonfat dry milk (Lab Scientific) or 5% bovine serum albumin, and incubated with the appropriate primary antibody overnight. The next day, secondary antibody was applied, and the signal visualized on a Molecular Imager ChemidocXRS (Bio-Rad Laboratories) using the Pierce Supersignal West Pico chemiluminescent substrate (Thermo Scientific). Images were obtained using Quantity One software (Bio-Rad Laboratories).

### Chromatin immunoprecipitation assays

Chromatin immunoprecipitation assays were performed as previously described [37]. Briefly, MCF-7 cells were crosslinked with 1% formaldehyde in PBS. For the precipitations protein A Dynabeads (10003D, Invitrogen) were coated with ERα antibody (#sc-8002 F-10) prior to pulldown and excess antibody was washed away. Pulldowns occurred while rotating for 16 h at +4C. Beads were then washed followed by elution from the beads using elution buffer (0.1 M NaHCO3, 1% SDS). Elutions were subsequently de-crosslinked overnight at +65C and DNA was purified using QIAquick PCR Purification Kit Protocol and used for QPCR.

### DNA-binding ELISA assay

To measure NFĸB family member DNA binding, and ELISA assay was performed using TransAM® NFĸB Family Activation Assay Kit (#43,296) following manufacturer’s protocol. Briefly, nuclear extracts of MCF-7 cells cultured in 2D and MS conditions were loaded on oligonucleotide coated plate and incubated for 1 h at room temperature, following by incubation with primary antibodies and secondary antibodies. For quantification of results, a spectrophotometer (BIO-TEK Synergy HT) was used within 5 min at 450 nm with a reference wavelength of 655 nm.

### Tumor initiation study

An in vivo tumor initiation study was carried out at the University of Illinois at Chicago animal facility and conducted in accordance with institutional procedures and guidelines, and after prior approval from the Institutional Animal Care and Use Committee. Female athymic nude mice (nu/nu), aged 5 week-old, were purchased from Harlan. Different numbers of MCF-7 cells grown in 2D or MS conditions were injected orthotopically into the thoracic mammary glands of mice supplemented with estrogen pellets. Three mice per group were used, each animal received 2 injections (*n* = 6). Tumor formation was monitored by palpitation for the next 6 weeks. ELDA (Extreme Limiting Dilution Analysis) was performed to calculate a significance difference of stem cell frequency for each group (*P* < 0.05) [38].

### Single-cell RNA sequencing (scRNA-seq) and data analysis

For scRNA-seq, MS were collected, washed twice with 1 × PBS, trypsinized and resuspended in × 1 PBS with 0.04% BSA. Cell suspension was loaded on a Chromium Single Cell 3′ Chip (10X Genomics). A detailed protocol of single-cell libraries preparation, sequencing process, raw data analysis and downstream analysis has been previously described [32]. Briefly, the standard workflow from Seurat package (Version 4.0.6) in the RStudio (v.4.0.3) was used for downstream analysis. Cell duplicates, cells with low gene counts (< 2000 genes), and cells with high mitochondrial gene expression (> 15% of total mapped reads) were excluded from analysis. This resulted in 2974 MCF-7 and 2765 T47D MS cells. Data will be available through Gene Expression Omnibus (GSE 205415) upon publication. To visualize the data in low-dimensional space, the Uniform Manifold Approximation and Project (UMAP) reduction technique was used. Cell cycle scoring and regression was performed by applying the cell cycle vignette from the Seurat package. For the integration of data from MS MCF-7 cells with data from 2D-cultured MCF-7 cells [39], from 4OHT-treated MCF-7 cells [32], or from long-term estrogen deprived (LTED) cells [40], we used publicly available dataset downloaded from Gene Expression Omnibus (GSE114462, GSE181812, GSE122743, respectively). Integration of these datasets was performed using the SCTransform vignette in the Seurat package to reduce technical variation caused by different methods of sample processing, as recommended by Hafemeister and Satija [41].

### Functional enrichment analysis

FEA was used to identify enrichment of gene signatures across the identified clusters as described in [40]. Signatures tested were derived from MSigDB v.7.4 [42, 43]. Prior to calculating signature scores, the data were normalized and scaled gene-wise. Then a z-scored signature was calculated for each cell separately. ROC analysis was used to estimate the accuracy of enrichment of a signature within a particular cluster. Area Under the Curve (AUC) > 0.6 was considered an enrichment. Significance of a signature enrichment across the clusters was estimated by the Wilcoxon rank-sum test (*P* < 0.01 was considered significant). The Pearson’s correlation coefficient and statistical significance were calculated using RStudio. Correlation coefficients of 0.3–0.5 indicate a moderate correlation and 0.5–0.9 indicate a strong correlation. Multiple linear regression was calculated by *lm* function in RStudio to establish the relationship between gene signatures in a specific cluster. The output data of analysis were downloaded using *library(broom)*. FEA for the MCF-7 MS Cluster 1 Signature was performed on scRNA-seq datasets from primary ER+ breast cancer tumors (GSE176078) [44], primary and metastatic patient derived xenograft (PDX) tumors with high (UCD4) and low (UCD46) expression of ER [45] (GSE131007) and on a scRNA-seq dataset from peripheral blood mononuclear cells derived from patients with metastatic breast cancer with Cluster 13 consisting of Circulation Tumor Cells (CTCs) (GSE174463) [46].

### Ingenuity pathway analysis (IPA)

The IPA package (QIAGEN Redwood City, www.qiagen.com/ingenuity) was used to identify a network connecting DEGs from the MS Cluster 1. The network and the type of connection between DEGs were formed based on the Ingenuity Knowledge Base repository inferred from the scientific literature [47].

### Statistical analysis

Data are presented as mean ± SEM from at least three independent determinations. Statistical analysis consisted of 1- or 2-way ANOVA followed by Tukey posttest, or t-test, as appropriate.

### Public data mining

Since the METABRIC dataset has a largest cohort of ER+ breast tumors (1175 patient tumors), it was chosen for estimation of predictive value of the MS Cluster 1 Signature. To access the METABRIC [Molecular Taxonomy of Breast Cancer International Consortium] cohort, cBioPortal for Cancer genomics, an open access resource providing a tool to analyze patient tumor samples was used [48, 49]. Genes from the MS Cluster 1 Signature were individually analyzed in the database against samples from the METABRIC cohort. Samples were stratified into two groups based on z-scored expression of each gene in the signature and named positive ( +) or negative (−) for signature alterations. Precomputed by cBioPortal z-scores were used for the analysis with the default setting (2 standard deviations from the mean). Overall survival, molecular subtype (PAM50 and claudin-low), neoplasm histological grade, and patient’s vital status were analyzed between the altered and unaltered groups according to the cBioPortal's online instructions and statistical significance was determined by chi-squared test. Calculation of the MS Cluster 1 signature score of the METABRIC cohort stratified for molecular subtype (PAM50 and claudin-low), neoplasm histological grade, and patient’s vital status was performed using RStudio, and mRNA expression matrix for each patient was downloaded from cBioPortal.

## Results

### scRNA-seq reveals a unique quiescent population of stem-like cells in ER+ mammospheres

**Fig. 1:**
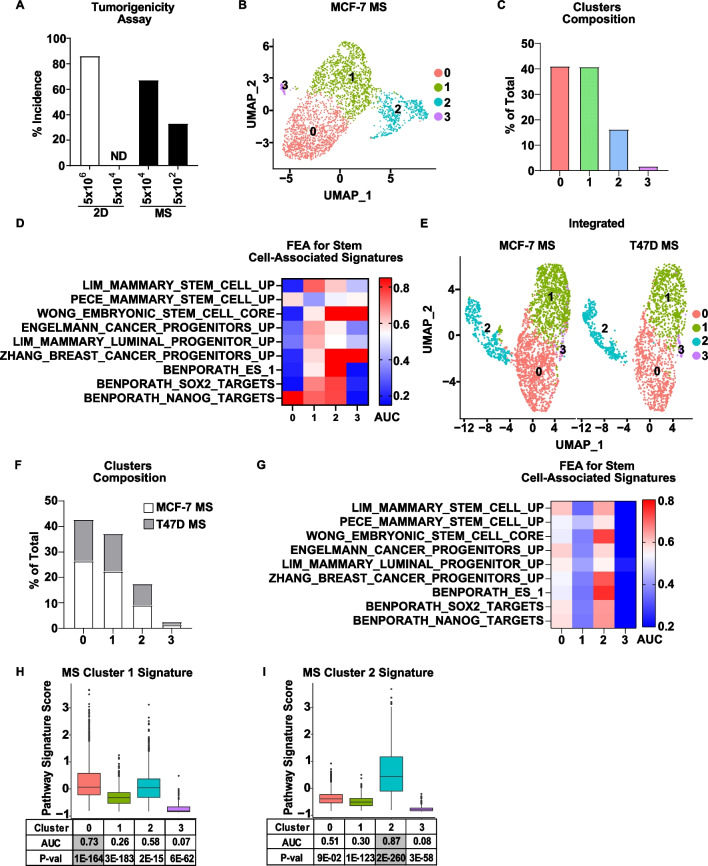
Single-cell RNA sequencing reveals two putative stem-like cell population clusters in mammospheres derived from ER+ breast cancer cell lines. **A** Different numbers of MCF-7 cells, cultured in standard 2D or mammosphere conditions, were injected into the mammary gland of athymic mice. The incidence of tumor formation was determined over 6 weeks. A significant difference in stem cell frequency, based on the tumor incidence per condition, was determined by ELDA (*P* < 0.05). ND, not detected. **B** scRNA-seq was conducted on MS derived from MCF-7 cells. Bi-dimensional representation of 2974 single-cell transcriptomes is shown (UMAP). **C** The percentage of cells in each cluster relative to the total number of cells is shown. **D** FEA was performed for stem cell-associated gene signatures from MsigDB. AUC values are shown in a heatmap, and *P*-values are presented in Additional file 2: Supplemental Table 3. **E** 5739 single-cell transcriptomes derived from MCF-7 MS and T47D MS were integrated and represented bi-dimensionally. **F** The distribution of MCF-7 and T47D cells in each cluster relative to the total cell number is shown. **G** FEA for stem cell-associated gene signatures was performed on the MCF-7 and T47D integrated dataset**.** AUC values are shown in a heatmap, and *P*-values are presented in Additional file 2: Supplemental Table 4. **H**, **I** FEA was performed for the gene signatures derived from the DEGs of MCF-7 MS Cluster 1 (**H**) and MCF-7 MS Cluster 2 (**I**) Signatures on the MCF-7 and T47D integrated dataset. Box plots show signatures scores per integrated cluster with significant AUC and *P*-values indicated in grey

**Fig. 2:**
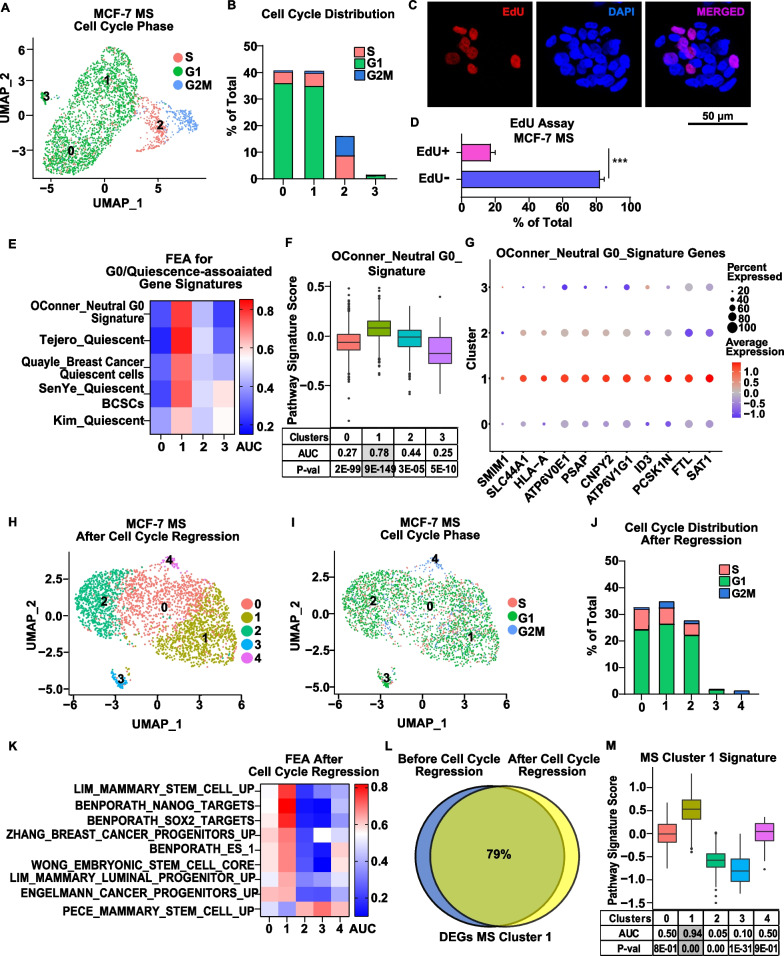
MS Cluster 1 is a quiescent stem-like cluster. **A**, **B** Cell cycle analysis was performed on MCF-7 MS using the Cell Cycle Scoring vignette provided by Seurat.** A** Bi-dimensional representations of 2974 single-cell transcriptomes colored by cell cycle phase (UMAP) and **B** cell cycle distribution for each cluster are shown. **C** An EdU assay was performed on MCF-7 MS. Representative pictures of MS stained for EdU (red) and DAPI (blue) are shown (bar = 50 µm) (**D**) A bar chart of EdU+ and EdU- cells presented as a percent of total number of cells from 11 MS is shown. **E**, **F** FEA was performed on the MS MCF-7 dataset with G0/quiescence-associated gene signatures with one representative example shown in a box plot. A detailed description of signatures is presented in Additional file 2: S. Table 6.1. AUC values are shown in a heatmap, and *P*-values are presented in Additional file 2: Supplemental Table 6.2. **G** Expression of genes associated with G0/Quiescence are represented in dot plots, with color representing expression level and size representing the percentage of cells in the cluster expressing the gene. **H** Cell cycle regression was performed using the Cell Cycle Regression vignette by Seurat. **I**, **J** Cell cycle phase and distribution are indicated after cell cycle regression of the MCF-7 MS dataset. **K** FEA was performed on MS MCF-7 dataset after cell cycle regression for stem cell-associated gene signatures from MsigDB. AUC values are shown in a heatmap, and *P*-values are presented in Additional file 2: Supplemental Table 7. (**L**) A Venn diagram shows percent of common DEGs derived from the MS Cluster 1 before and after cell cycle regression. **M** FEA was performed for the original MCF-7 MS Cluster 1 Signature on the MS MCF-7 dataset after cell cycle regression

**Fig. 3:**
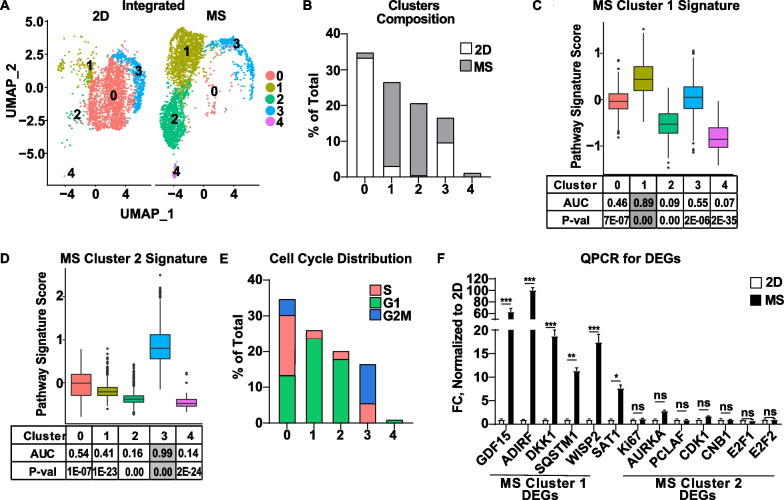
Integration of single-cell RNA sequencing datasets from MCF-7 cells cultured in standard 2D conditions and MS. **A** 5592 single-cell transcriptomes derived from 2D- and MS-cultured MCF-7 cells were integrated and represented bi-dimensionally. **B** The distribution of 2D- and MS-cultured MCF-7 cells in each cluster relative to the total cell number is indicated. **C**, **D** FEA was performed for the gene signatures derived from the DEGs of the original MS Cluster 1 (**C**) and MS Cluster 2 (**D**) on the integrated dataset. **E** Cell cycle distribution for each cluster as the percent of total cell number is indicated. **F** QPCR for the top DEGs of MS Cluster 1 and MS Cluster 2 was performed in MCF-7 cells cultured in 2D vs MS conditions. Data are presented as fold change (FC) normalized to 2D cells on a per gene basis. *P < 0.05, **P < 0.005, ***P < 0.001, *ns* not significant

To study BCSCs, we isolated cells from mammospheres (MS), which we and others [35, 36] have shown are highly tumorigenic compared to 2D-cultured cells (Fig. 1A), and thus are enriched for BCSCs. We performed scRNA-seq on cells derived from MS of two ER+ breast cancer cell lines, MCF-7 and T47D, and then assessed BCSC properties using several widely recognized characteristics of stem cells: i) expression of classical stem cells markers and an enrichment for stem cell-associated gene signatures (Fig. 1), ii) proliferation status (Fig. 2 and Additional file 1: S. Fig 1), and (iii) enrichment in MS vs 2D-cultured cells (Fig. 3).

We first performed unsupervised clustering of cells derived from MCF-7 MS, which revealed 4 cell clusters with distinct transcriptomes (Fig. 1B, C). Analysis of classical stem marker expression across the 4 clusters revealed that: (i) expression of some stem markers was not detected in our dataset (e.g., *ALDH1A1* and *KRT5*), (ii) some markers were expressed but to a similar level among the four clusters, (e.g., *CD44, NANOG, POU5F1/Oct4*), and (iii) some markers were differentially expressed across the clusters but not in the same cluster (e.g., *CD24* and *TWIST* were expressed in Cluster 0, *KLF4* was expressed in Cluster 1, Additional file 2: S.Table 1). Since cellular processes are often associated with changes in the expression of groups of genes that share common biological functions or properties, we reasoned that a more reliable approach to identify putative BCSCs will be to test a set of related genes (i.e., a gene signature) rather than rely on expression of any single gene, which could be affected by technical limitations of scRNA-seq, such as gene dropout [50]. For this purpose, we used MsigDB [42] as a source of stem cell-associated gene signatures, from which we selected 9 signatures that have been derived from or validated in in breast cancer models (a detailed description of each signature is presented in Additional file 2: S. Table 2). To identify which cluster(s) are enriched for stem cell-associated gene signatures, we applied Functional Enrichment Analysis (FEA), also known as Gene Set Enrichment Analysis, a widely used approach which was proven to detect even small but biologically significant changes in gene expression in different cancer datasets [40, 51]. We found that the majority of these stem cell-associated gene signatures were significantly enriched in MS Cluster 1 and MS Cluster 2 (Fig. 1D, Additional file 2: S. Table 3), suggesting that these clusters may be putative stem-like cell populations. To determine if similar putative stem-like populations are found in T47D MS, we performed an integration analysis of MCF-7 and T47D MS datasets, which resulted in 4 clusters, each consisting of cells from both lines (Fig. 1E, F), indicating transcriptomic similarities between MCF-7 MS and T47D MS. Two clusters of the integrated MCF-7/T47D dataset, Integrated Cluster 0 and Integrated Cluster 2, were found to be enriched for stem cell-associated gene signatures (Fig. 1G, Additional file 2: S. Table 4). To establish whether these two clusters are similar to the two stem-like clusters originally identified in MCF-7 MS, we used FEA for custom gene signatures generated from differentially expressed genes (DEGs) (Additional file 2: S. Tables 5.1–5.4) of MCF-7 MS Cluster 1 and Cluster 2 (Additional file 2: S. Tables 5.2, 5.3) (hereinafter referred to as “the MS Cluster 1 Signature” and “the MS Cluster 2 Signature”). FEA showed enrichment for the MS Cluster 1 Signature in the Integrated MCF-7/T47D Cluster 0, and the MS Cluster 2 signature was found to be enriched in the Integrated MCF-7/T47D Cluster 2 (Fig. 1H, I and Additional file 1: S. Fig 1). These results indicate that MS derived from different ER+ breast cancer cell lines have 2 common cell populations with stem-like gene signature enrichment.

We next focused on cell proliferation, since some studies have shown that stem cells remain primarily in a quiescent/low proliferative state to maintain their stemness [23, 52]. To understand which of the two MS stem-like populations are quiescent, we first performed a cell cycle scoring analysis. The majority of MS Cluster 1 cells were found to be in G1 cell cycle phase whereas MS Cluster 2 cells were split evenly between S and G2M (Fig. 2A, B). We observed a similar cell cycle distribution for the two putative stem-like populations in the integrated MCF-7/T47D dataset (Additional file 1: S. Fig 2). Next, we performed a functional EdU incorporation assay, which supported our bioinformatic analysis and showed a similar distribution of proliferative vs non-proliferative cells in MS for both cell lines (Fig. 2C, D and Additional file 1: S. Fig 2C, D). To identify which cluster is quiescent, we next utilized gene signatures derived specifically from cells in G0/quiescent state (a detailed description of signatures presented in Additional file 2: S. Table 6.1). FEA for these gene signatures showed enrichment exclusively in MCF-7 MS Cluster 1 (Fig. 2E–G) and in Cluster 0 of the integrated MCF-7/T47D dataset, indicating the presence of quiescent cells in these populations (Additional file 1: S. Fig 2 E–G, Additional file 2: S. Table 6.2).

Previously, Ben-Porath et al. showed that stem cell-associated signatures may inherit proliferation-related genes, which may explain the enrichment of both proliferative cells and stem cell-associated gene signatures in MS Cluster 2 [53]. Additionally Ben-Porath et al. showed that regression of proliferation-related genes from analysis did not affect stem cell-associated gene signature enrichment [53]. Based on these concepts, we decided to conduct cell cycle regression to eliminate the influence of proliferation from the MS dataset and re-cluster the cells to determine whether stem cell-associated gene signature enrichment was reliant on or independent of proliferation-associated genes (Fig. 2H–J). We found that only one cluster, Cluster 1, was predominantly enriched for stem cell-associated signatures after cell cycle regression (Fig. 2K, Additional file 2: S. Table 7). This cluster has up to 80% of genes in common with the original MS Cluster 1 identified before cell cycle regression and was enriched for the MS Cluster 1 Signature (Fig. 2L, M). However, no enrichment of the original MS Cluster 2 Signature was observed in this population (data not shown). These findings indicate that enrichment for stem cell-associated signatures in MS Cluster 1 is independent of proliferation-related genes and retained even after cell cycle regression, while enrichment for stem cell-associated gene signatures in MS Cluster 2 completely relies on the expression of proliferation-related genes.

Since it is well established that MS are enriched for BCSCs, we next examined whether either of the 2 putative stem-like populations are enriched in MS. To do this, we integrated the MCF-7 MS dataset with a dataset from MCF-7 cells in standard 2D culture (MS/2D integrated dataset) [39]. Unsupervised clustering revealed 3 clusters that are highly enriched for MS cells: Cluster 1, Cluster 2, and Cluster 4 (Fig. 3A, B). FEA revealed that the original MS Cluster 1 Signature is enriched in the MS/2D Integrated Cluster 1 (Fig. 3C), which consists primarily of MS cells. In contrast, the MS Cluster 2 Signature was enriched specifically in the MS/2D Integrated Cluster 3, which consists of both MS- and 2D-cultured cells (Fig. 3D and Additional file 1: S. Fig 3). As expected, cells from MS/2D Integrated Cluster 1 were predominantly in G1 cell cycle phase, consistent with quiescence, whereas cells from MS/2D Integrated Cluster 3 were found to be in S/G2M cell cycle phases (Fig. 3E), indicating this proliferative population can be found in both MS- and 2D-cultured cells. Using QPCR, we confirmed that MS Cluster 1 DEGs are in fact elevated in MS compared to 2D-cultured cells, while MS Cluster 2 DEGs were not different between MS and 2D (Fig. 3F). Collectively, these findings suggest that MS Cluster 1 represents a putative BCSC population based on its expression of stem cell-associated gene signatures, quiescence, and enrichment in MS.

### ER and NFĸB are active in MS Cluster 1 and required for MS formation

**Fig. 4:**
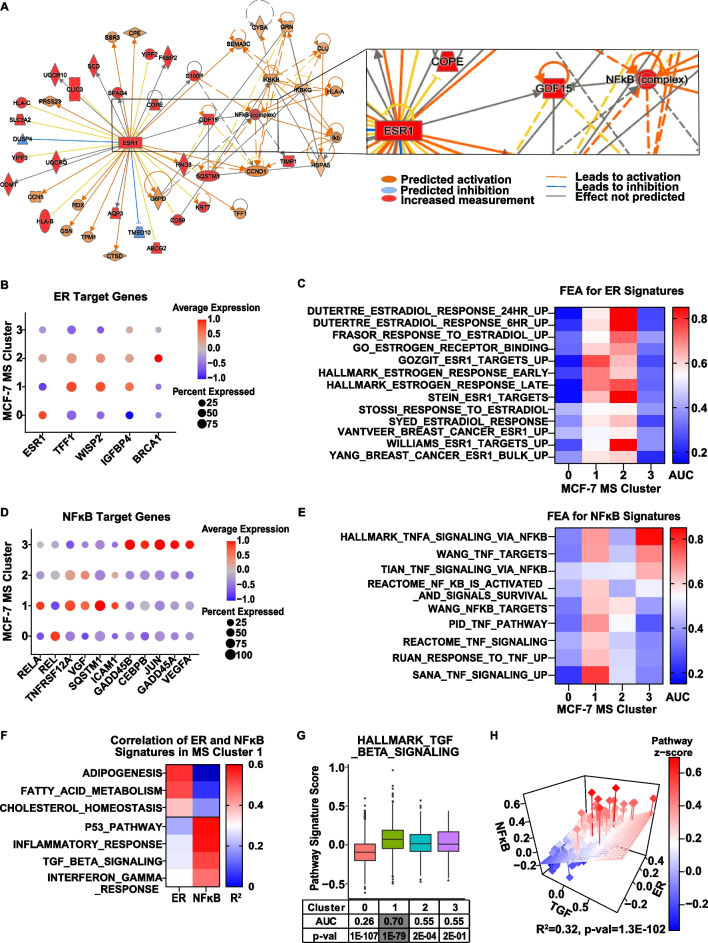
ER and the NFĸB pathway are active in MS Cluster 1 and required for MS formation. **A** IPA network analysis was performed for DEGs of the original MS Cluster 1. Two central nodes were identified, *ESR1* (*P*-val = 4.73E-9) and NFĸB complex (*P*-val = 3.72E-3). Expression of *ESR1* and ER target genes (**B**) and NFĸB family members and NFĸB target genes (**D**) are presented in dot plots, with color representing expression level and size representing the percentage of cells in each cluster expressing the gene. FEA was performed for ER (**C**) and NFĸB (**E**) gene signatures from MsigDB. AUC values are shown in a heatmap, and *P*-values are presented in Additional file 2: Supplemental Table 8. **F** Correlation between Hallmark ER or Hallmark NFĸB signatures with other Hallmark gene signatures in MS Cluster 1 was calculated using Pearson’s Correlation function in RStudio software. Correlation values are shown in a heatmap and *P*-values are presented in Additional file 2: Supplemental Table 9. **G** FEA was performed for the Hallmark TGFβ pathway gene signature on MCF-7 MS Clusters. Box plot shows signature scores per cluster and table indicates AUC and *P*-values for each cluster. **H** Multiple linear regression analysis between Hallmark ER, NFĸB and TGFβ pathway gene signatures was performed for MS Cluster 1. Single cells individually placed in 3D graph with each axis representing the z-score for each pathway and each rectangle representing individual cells

To identify which genes/pathways are active and may promote stem properties in MS Cluster 1, we performed IPA network analysis on DEGs of this cluster (Fig. 4A). We found that top upregulated DEGs form a network with two central nodes, ESR1 and NFĸB complex, suggesting that both are active in MS Cluster 1. To assess this, we examined expression of ER and NFĸB family members, as well as ER and NFĸB activity based on target gene expression and target gene signature enrichment in the original MS Clusters. We found that ESR1 was most highly expressed in MS Clusters 0 and 2, whereas expression of ER target genes and enrichment of ER-associated gene signatures was observed in MS Clusters 1 and 2, suggesting that ER is active in MS Clusters 1 and 2 (Fig. 4B, C and Additional file 2: S. Table 8). NFĸB family members, RELA and REL, are highly expressed in MS Clusters 1 and 0, respectively, while expression of NFĸB target genes and enrichment of NFĸB-associated gene signatures were found in MS Clusters 1 and 3 (Fig. 4D, E and Additional file 2: S. Table 8). These findings confirm the IPA analysis indicating that both ER and NFĸB are active in MS Cluster 1.

To understand the function of ER and/or NFĸB in MS Cluster 1, we utilized the IPA network analysis to identify MS Cluster 1 DEGs that can potentially be regulated by ER or NFĸB independently, or by the two working together, interdependently. For independent mechanism(s), we performed a correlation analysis between Hallmark pathways in MsigDB and found that the HALLMARK_ESTROGEN_RESPONSE_LATE gene signature correlates with several lipid metabolism-associated pathways, whereas HALLMARK_TNFA_VIA_NFKB gene signature correlates primarily with inflammatory and immune response pathways (Fig. 4F and Additional file 2: S. Table 9). These findings correspond with previously published data showing ER and cholesterol pathways are associated with BCSC maintenance [54, 55], while NFĸB promotes stem properties by regulating pro-inflammatory cytokines [56]. As these pathways have been shown previously to be involved in regulation of stem cells, we decided to focus MS Cluster 1 DEGs that can be regulated by both ER and NFĸB working together. We found that *GDF15* (growth/differentiation factor-15), a top 10 DEG for MS Cluster 1 (Additional file 2: S. Table 5.2), was predicted to be upregulated by both ER and NFĸB (Fig. 4A insert). *GDF15* is a member of the TGFβ signaling pathway and has been shown to play role in promotion of stem-like features in multiple cancers [57–59]. In breast cancer cell lines, a recent study by Sasahara et al. showed that *GDF15* treatment increases expression of stem-associated genes and mammosphere forming efficiency, which was reversed by a *GDF15* blocking antibody [57]. Since *GDF15* is a member of the TGFβ superfamily, we performed FEA for a TGFβ pathway gene signature and found it to be enriched in MS Cluster 1 (Fig. 4G). Moreover, we observed a correlation between the expression of the hallmark TGFβ gene signature and both the Hallmark ER and NFĸB gene signatures in MS Cluster 1 cells (Fig. 4H). These findings suggest that ER and NFĸB may promote stem-like features in MS Cluster 1 through regulating of *GDF15* and activation of TGFβ signaling.

### ER and NFĸB work together to promote stem-like features through GDF15

**Fig. 5:**
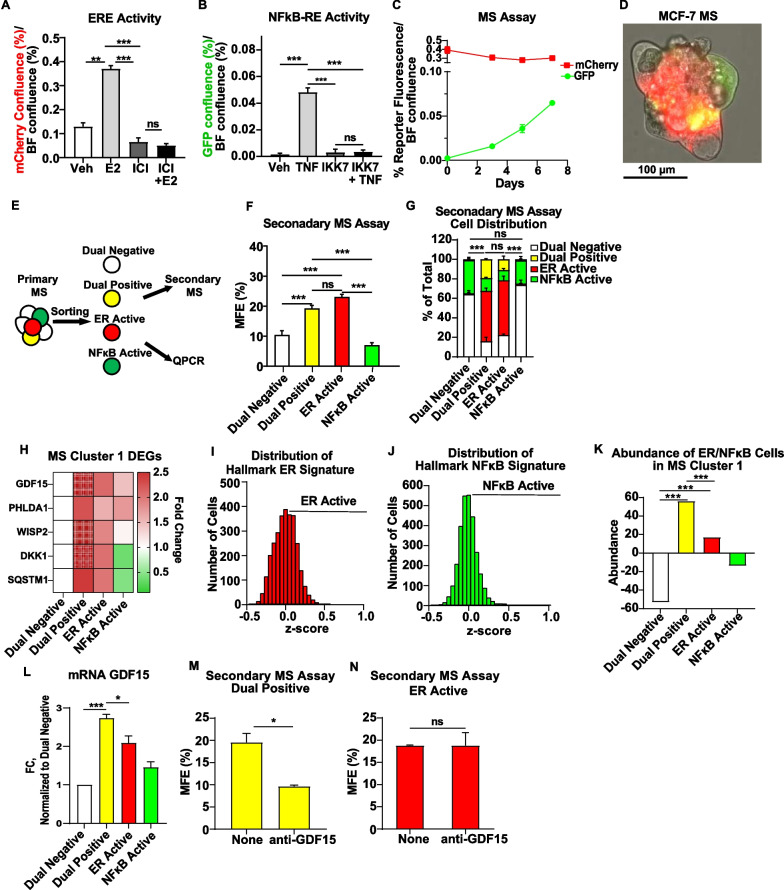
Dual reporter cell lines identify an ER and NFĸB-driven stem-like cell population. **A**, **B** ERE-mCherry and NFĸB-RE-eGFP activity in 2D-cultured MCF-7 dual reporter cells treated with E2 (10 nM), hTNFα (10 ng/ml), ICI (1 µM) and/or IKK7 (1 µM) for 24 h was measured using a Celigo imaging cytometer. Bar charts represent the percentage of mCherry confluence (**A**) and eGFP (**B**) confluence normalized to total brightfield confluence for each treatment group. **C** ERE-mCherry and NFĸB-RE-eGFP activity in MCF-7 MS was measured over time. **D** A representative image of MS derived from MCF-7 dual reported cell line is shown (bar = 100 µm). **E** A schematic of sorting experiments performed on MS derived from the MCF-7 dual reporter cells is presented. **F** MS derived from MCF-7 dual reporter cells were collected, trypsinized and sorted for 4 cell populations, based on expression of fluorescent proteins eGFP and mCherry. A secondary MS assay was performed on 4 sorted populations and secondary MFE for each group is plotted. **G** Cell distribution of secondary MS is plotted for each group based on ERE-mCherry and NFĸB-RE-eGFP activity. **H** The expression of DEGs of MS Cluster was determined in each cell population by QPCR. Fold change, normalized to dual-negative cell population, for each gene is presented on a heatmap. **K**, **L** Distribution of z-scores for the Hallmark ER (**I**) and NFĸB (**J**) signatures are shown on a per cell basis. **K** Each cell from MS Cluster 1 was assigned to one of the groups based on z-scores for ER and NFĸB signatures: dual-negative cells (white), dual-positive cells (yellow), ER-active cells (red), NFĸB-active cells (green). The percent change in abundance of MS Cluster 1 relative to the total population for each group is shown. **L** A bar charts representing GFD15 mRNA expression in each sorted group in (**K**)**. M**, **P** The role of GDF15 on secondary MS formation was examined using an anti-GDF-15 antibody (200 ng/ml) on 2 sorted cell population from: dual-positive (**M**) and ER-active (**N**). *P < 0.05, ***P < 0.001, *ns* not significant

To validate our bioinformatic findings, we assessed ER and NFĸB activity in bulk MS cells (Additional file 1: S. Fig 4) and in isolated cell populations (Fig. 5, Additional file 1: S. Fig 5). In bulk MCF-7 and T47D MS, we found elevated ER and NFĸB activity based on target gene expression (Additional file 1: S. Fig 4A, 4E), DNA binding activity (Additional file 1: S. Fig 4B, 4F) and/or nuclear localization compared to 2D culture (Additional file 1: S. Fig 4C, 4G). To understand if ER and NFĸB activity are necessary for MS formation, we examined MS forming efficiency (MFE) in the presence of ICI182,780, an ER antagonist, and IKK7, an inhibitor of upstream kinases in the NFĸB pathway, IKKα/β. We found that each inhibitor reduced MFE in a dose-dependent manner (Additional file 1: S. Fig 4D, 4H), but that sublethal doses of ICI and IKK7 substantially decreased MFE compared to either inhibitor alone (Additional file 1: S. Fig 4I, 4J). These findings were further confirmed in 2 new ER+ breast cancer cell lines derived from patient-derived xenografts (PDX), UCD4 and UCD65 (Additional file 1: S. Fig 4K, 4L). These findings suggest that while both pathways are required, they may also work together cooperatively, as we have previously shown on target genes, MS formation, and BCSC genes [33, 60]. We also confirmed that GDF15 is regulated by ER and NFĸB to some extent in the bulk MCF-7 MS (Additional file 1: S. Fig 4M).

While these findings support a role for ER and NFĸB in MS in general, a population-specific approach is needed to uncover the role of ER and NFĸB in MS Cluster 1’s stem-like properties. Thus, in order to isolate MS Cluster 1 cells, we decided to take advantage of their ER and NFĸB activity. We established dual reporter ER+ breast cancer cell lines, where activation of NFĸB is detected by eGFP expression and activity of ER is indicated by mCherry expression. We confirmed that expression of fluorescent proteins corresponds to ER and NFĸB activation by E2 and TNFα, and that this activation is inhibited by ICI and IKK7, respectively (Fig. 5A, B and Additional file 1: S. Fig 5A, 5B). As expected, we found ER and NFĸB to be active in MS, with constant ER activity throughout MS development, while NFĸB activity was low initially but increased over time (Fig. 5C and Additional file 1: S. Fig 5C). In fully developed MS, 4 populations of cells with heterogeneous ER and NFĸB activity were observed: dual-negative (white), dual-positive (yellow), ER-active (red), and NFĸB-active (green) (Fig. 5D and Additional file 1: S. Fig 5D).

We next performed a sorting experiment to examine MS formation efficiency (MFE) and expression of the MS Cluster 1 DEGs (Fig. 5E) for each population. We found that MFE (Fig. 5F, Additional file 1: S. Fig 5E) were higher in two cell populations ER-active and dual-positive. Additionally, both of these populations showed similar abilities to form secondary MS with all 4 progeny: dual-negative, dual-positive, ER-active and NFĸB-active (Fig. 5G, Additional file 1: S. Fig 5F), suggesting that ER-active and dual-positive cell populations are not stable and able to repopulate a heterogenous MS. Along with MFE, expression of the MS Cluster 1 DEGs (Fig. 5H) were higher in the same two cell populations, ER-active and dual-positive. Taken together these findings suggest that MS Cluster 1, does not consist of dual-positive cells exclusively and may be distributed across both the ER-active and the dual-positive cell populations. To test this, we estimated the enrichment of MS Cluster 1 with each cell population based on ER and NFĸB activity. To calculate the enrichment, we first determined activation of ER and NFĸB pathways using z-scores of Hallmark NFĸB and ER gene signatures for each cell individually from MS Cluster 1 as an alternative approach to FEA for the entire cluster (Fig. 5I, J). We next used these signatures’ z-scores to assign each cell to a specific population (dual-negative, dual-positive, ER-active or NFĸB-active). Finally, enrichment for dual-positive, dual-negative, ER-active and NFĸB-active cells in MS Cluster 1 was calculated (Fig. 5K). Indeed, this approach showed that MS Cluster 1 is enriched for cells with high z-scores for both ER and NFĸB signatures and for ER signature alone, confirming the presence of both dual-positive and ER-active cell subpopulations in MS Cluster 1. However, the abundance of the dual-positive cells was significantly higher compared to the ER-active cells in MS Cluster 1 (Fig. 5K). The higher expression of *GDF15* compared to other populations (Fig. 5L) suggested its involvement in the regulation of stem properties specifically in the dual-positive cells. To test this, we isolated and treated two cell populations, dual-positive and ER-active, with an antibody that inhibits GDF15 (anti-GDF15). It was found that inhibition of *GDF15* reduces MS formation in the dual-positive cell population, but not in the ER-active cell population (Fig. 5M, N). These data suggest that the functional role of *GDF15* in promotion of stem properties depends on activity of both ER and NFĸB and is restricted to a specific subpopulation of the MS Cluster 1 cells.

### A gene signature derived from MS Cluster 1 is expressed in endocrine resistant and metastatic cell populations and is associated with aggressive disease.

**Fig. 6:**
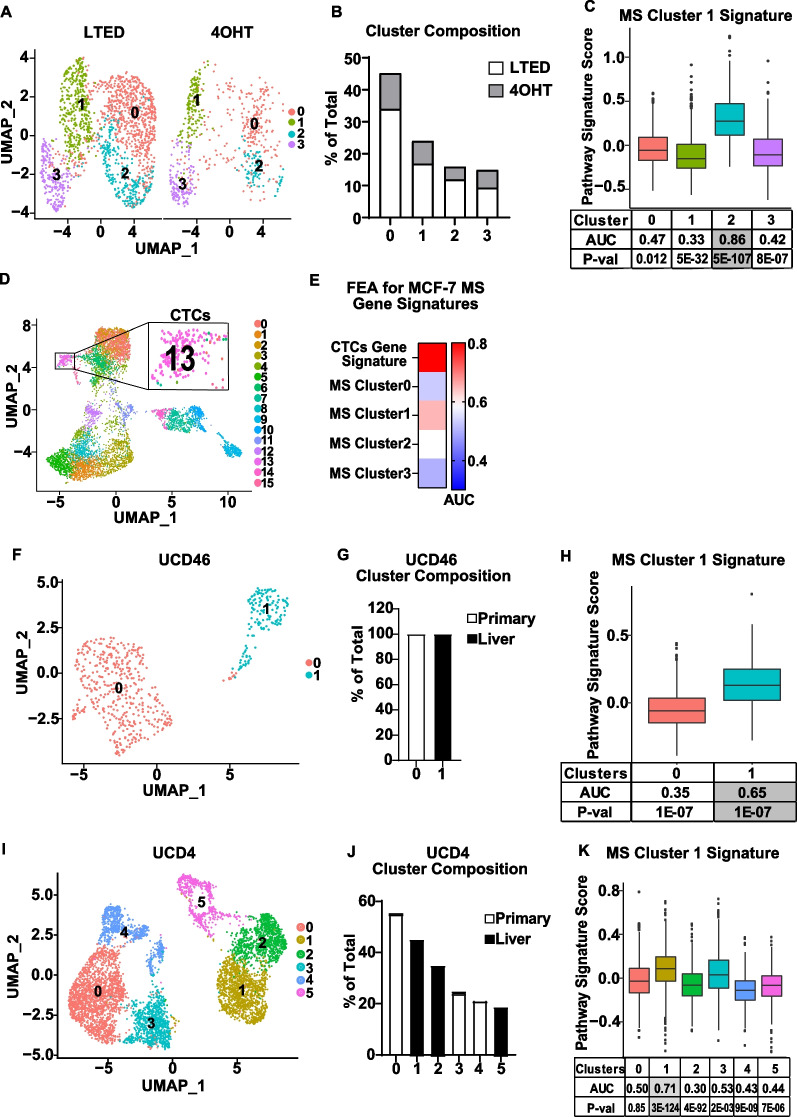
The MS Cluster 1 signature is associated with more aggressive metastatic disease. A Single-cell transcriptomes from 4OHT-treated MCF-7 cells (GSE181812) and LTED (Long-Term Estrogen Deprived) MCF-7 cells (GSE122743) were integrated and represented bi-dimensionally. **B** The distribution of 4OHT-treated MCF-7 cells and LTED MCF-7 cells in each cluster relative to the total cell number is shown. **C** FEA was performed on integrated datasets for the MS Cluster 1 Signature. **D** Bi-dimensional representation of 6519 single-cell transcriptomes of peripheral blood mononuclear cells derived from patients with metastatic breast cancer are shown (UMAP) with Cluster 13 presenting of Circulation Tumor Cells (CTCs) (GSE174463). **E** FEA was performed for the custom genes signatures derived from DEGs of each MS Cluster on CTCs Cluster 13. AUC values are shown in a heatmap, and *P*-values are presented in Additional file 2: Supplemental Table 9. **F**, **I** Single-cell transcriptomes from primary and metastatic tumors of PDX models UCD46 (**F**) and UCD4 (**I**) are represented in UMAP plots (GSE131007). **G**, **J** The proportion of cells in each cluster is indicated by their origin (i.e., primary tumor or metastatic location) relative to the total number of cells of each origin. **H**, **K** FEA was performed for the MS Cluster 1 Signature on both datasets with box plots showing signature scores per cluster

Given the known role of BCSCs in therapy resistance, disease progression and metastasis, we next investigated whether MS Cluster 1 can be a predictor of aggressive ER+ disease. To answer this question, we took several approaches. First, we interrogated two integrated datasets derived from MCF-7 cells that are tolerant [32] or fully resistant to endocrine therapy [40] for expression of the MS Cluster 1 Signature (Fig. 6A, B and Additional file 1: S. Fig 6). We found that the signature was enriched in Integrated Cluster 2, which consists of tolerant and fully resistant cells (Fig. 6B, C), suggesting that the MS Cluster 1 is retained in a subpopulation of endocrine therapy tolerant/resistant cells. Each dataset was also tested individually and showed similar results (Additional file 1: S. Fig 6). We next investigated expression of the MS Cluster 1 Signature in Circulating Tumor Cells (CTCs) using a publicly available dataset of peripheral blood mononuclear cells derived from metastatic breast cancer patients [46]. Unsupervised clustering revealed 16 clusters, one of which represents CTCs (Cluster 13, Fig. 6D). FEA showed specific enrichment of the MS Cluster 1 Signature in the CTC population, suggesting the involvement of MS Cluster 1 cells in metastasis (Fig. 6E and Additional file 2: S. Table 10). To test this, we examined enrichment of the MS Cluster 1 Signature in primary vs. metastatic tumor cell populations. For that purpose, we utilized scRNA-seq datasets from primary and metastatic patient derived xenograft (PDX) tumors, UCD46 and UCD4, with low and high expression of ER, respectively. For the UCD46 PDX model, we identified 2 cell clusters and for UCD4 we identified 6 cell clusters (Fig. 6F, I), with each cluster consisting of a different ratio of primary or metastatic cells (Fig. 6G, J). FEA for the MS Cluster 1 Signature showed enrichment in Cluster 1 of UCD46, and Cluster 1 of UCD4, both of which consist of liver metastases (Fig. 6H, K), suggesting that MS Cluster 1 cells may be more abundant at metastatic sites than in primary tumors.

**Fig. 7:**
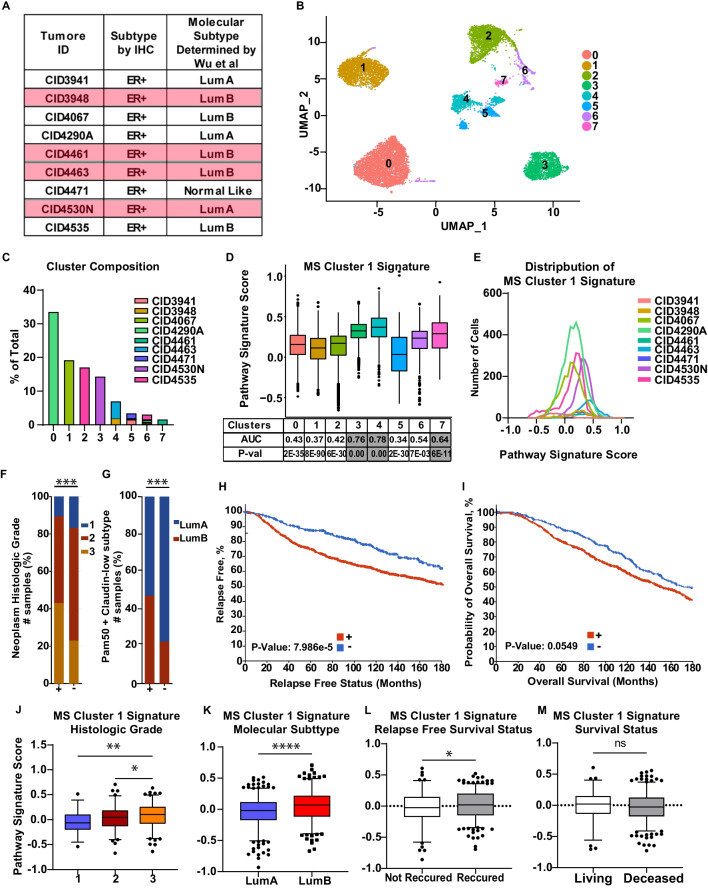
The MS Cluster 1 Signature is associated with poor patient outcome. **A** A description of nine ER+ breast tumors processed with scRNA-seq technologies which datasets were downloaded from GEO portal (GSE176078) ^44^. **B**, **C** 31,259 single-cell transcriptomes of epithelial cells from (**A**) were clustered and represented bi-dimensionally (**B**) with cluster compositions (**C**). **D** FEA was performed for the MS Cluster 1 Signature on ER+ breast cancer datasets (**A**–**C**) with box plot showing signature scores, AUC and *P*-value per cluster. **E** Distribution of z-scores for the MS Cluster 1 Signature is shown on a per cell basis for each tumor from (**A**). **F**–**I** The MS Cluster 1 Signature was interrogated in 1175 ER+ breast tumors from the METABRIC cohort available in cBioPortal for Cancer Genomics. Histologic grade (**F**), molecular subtype (**G**), patient relapse free survival (**H**) and overall survival (**I**) between tumors+ vs.— for expression of the MS Cluster 1 Signature are displayed. Statistical significance was determined using chi-squared test (**F**, **G**) or log-rank test (**H**, **I**). **J**–**M** The MS Cluster 1 signature score distribution across ER+ breast tumors from (**H**–I) stratified by histologic grade (**J**), molecular subtype (**K**), relapse free survival status (**L**), and survival status (**M**). *P*-values are from Student’s tests. *P < 0.05,**P < 0.01, ***P < 0.001, *ns* not significant

We next tested whether the MS Cluster 1 Signature enrichment can be detected in scRNA-seq datasets derived from primary ER+ breast cancer tumors [44] (Fig. 7A). We observed the same clustering pattern of cells by tumor identity, as shown by Wu et al. [44] (Fig. 7B, C). FEA showed that the MS Cluster 1 Signature was highly enriched in 3 clusters: Cluster 3, Cluster 4 and Cluster 7, which represent tumors CID4530N, CID4463, CID3948, and CID4461 (Fig. 7D). For other tumors, we found that individual cells demonstrate a high MS Cluster 1 signature score but that these cells did not drive clustering (Fig. 7E), so the overall signature score did not reach significance in any individual cluster. These data imply that cells expressing the MS Cluster 1 signature can be found in primary ER+ tumors to varying degrees, but in some tumors may only be identified at the single-cell resolution. Finally, we questioned whether the MS Cluster 1 Signature may be a predictor of poor outcome for breast cancer patients by interrogating a publicly available METABRIC dataset of ER+ tumors. It was found that tumors expressing the MS Cluster 1 Signature are more likely to be high grade, and of the Luminal B subtype, are associated with an increased risk of relapse, and showed a trend toward lower overall survival (Fig. 7F–I). Moreover, the higher signature score was observed in tumors with high histologic grade, Luminal B subtype and recurred tumors (Fig. 7J–M). Taken together, these findings indicate that the MS Cluster 1 Signature is associated with endocrine therapy resistance and metastases, and is predictive of poor patient outcome, implying that the MS Cluster 1 cell population represents a unique BCSC population that may be more abundant in aggressive ER+ disease.

## Discussion

ER+ breast tumors are known to be heterogeneous and harbor a subpopulation of cells with stem-like properties that can contribute to the development of therapy resistance, tumor metastasis, and poor patient survival. Here, we identified a novel ER-NFĸB-driven stem-like population derived from ER+ breast cancer cell lines grown as MS, and demonstrate that this population is clinically relevant as its gene signature is expressed in aggressive breast cancer phenotypes and is predictive of tumor relapse.

One major question arising from our studies is whether the MS Cluster 1 stem-like population is unique or related to known BCSC populations. We established that the MS Cluster 1 population is enriched for multiple stem cell-associated gene signatures, expands in MS conditions, and remains quiescent, but we did not observe the expression of classical stem cell markers. The lack of marker expression could be explained by technical limitations of scRNA-seq since it captures approximately 10–30% of transcripts per cell [61]. Thus, the absence of markers is not conclusive evidence that they are not expressed. In bulk MS, we did detect CD44+/CD24− and ALDH1+ cell populations by FACS analysis, as well as expression of stem cell-associated markers by QPCR (data not shown), confirming that MS is indeed enriched for classical BCSCs genes. Alternatively, it has been shown that classical stem makers, originally derived from triple-negative breast cancer, may not be applicable to ER+ BCSCs. For example, in a recent study, Li and colleagues compared the ratio of CD44+/CD24− and ALDH1+ cell populations across different breast cancer molecular subtypes and showed that MCF-7 cell line (Luminal A subtype) has the lowest ratio of both cell populations [62]. Additionally, the CD44+/CD24− cell population derived from MCF-7 cells showed lower tumorigenicity capacity compared to the CD44+/CD24− population derived from triple-negative breast cancer cell line, suggesting that CD44+/CD24− and ALDH1+ are excellent markers of BCSCs from ER- tumors but possibly less suitable markers for BCSCs of ER+ tumors [62]. These findings were supported by Coates and colleagues who argue for the development of a panel of markers specific for each breast cancer subtype rather than using the same markers for all breast tumors [63]. For these reasons we relied on functional characteristics of stem cells, such as MS formation and cell cycle analysis, in combination with multiple stem cell-associated gene signatures, to study the MS Cluster 1 stem-like population, which we speculate could be specific for ER+ tumors. We also attempted to isolate this population by sorting dual reporter cell lines based on ER and NFĸB activity, but we found that not all the cells in MS Cluster 1 had ER and NFĸB activity and this cluster consists of 2 cell subpopulations with stem properties. Thus, to answer whether this is a unique BCSC population an alternative approach to specifically isolate the MS Cluster 1 stem-like population is needed.

In this study, we also shed light on the controversial role of ER in promoting stem properties in ER+ breast cancer. As was described in the introduction, both ER activation and ER inhibition have been shown to promote expansion of BCSCs in ER+ breast cancers [13–22]. Here, we demonstrated that ER is required for the promotion of stem properties as its inhibition leads to the decrease of MS formation. Moreover, we showed that ER is active in the MS Cluster 1 stem-like population, as indicated by bioinformatics analysis and by reporter assays, and we argue that the expression of ER should be interpreted with caution since it does not always represent ER activity.

Along with ER, the NFĸB pathway was found to be active in the MS Cluster 1 stem-cell population. In the literature, expression of ER and NFĸB and their activity are often reported as inversely correlated, and this appears to be the case in BCSCs as well [8, 64]. For example, Jiang and colleagues identified a stem-like population with low ER expression and high NFĸB activity in mammary tumors of MMTV-PyMT mice [8]. Likewise, Gomes et al. showed that MCF-7 cells with overexpression of receptor activator of NFĸB (RANK) are characterized by downregulation of ER, upregulation of stem cell markers, and a higher MS formation capacity [64]. However, our recent work has suggested that ER and NFĸB can also work together to promote BCSCs. More specifically, we found that co-activation of ER and NFĸB increased mammosphere formation, as well as proportion of cells with BCSC markers [33]. Mechanistically, we demonstrated that ER and NFĸB work together to regulate a feedback loop involving the downregulation of miR-181 and the upregulation of its target, PHLDA1, to enhance stem properties in ER+ breast cancer cell lines [65]. In this study, we provide further evidence that these pathways can work together to promote BCSCs, as the MS Cluster 1 stem-like population consists primarily of dual-positive cells that are more MS forming and dependent on *GDF15*, a common ER and NFĸB target.

In addition to our biological findings, we showed that the MS Cluster 1 stem-like population is clinically relevant. We found expression of the MS Cluster 1 gene signature in primary human tumors is associated with worse clinicopathological features and reduced relapse free survival. Moreover, cell populations derived from endocrine tolerant and resistance cells, CTCs, and breast tumor metastases showed enrichment for the MS Cluster 1 Signature, implying that the stem-like population is abundant in more aggressive phenotypes and can contribute to their development. With the integration of scRNA-seq technology into the clinic, we expect that the MS Cluster 1 Signature will become increasingly useful in the prediction of ER+ disease progression and optimization of patients’ treatment by identifying the stem-like population within a tumor.

## Supplementary Information

**Additional file 1:** Supplementary Figures.

**Additional file 2:** Supplementary Tables.

## Data Availability

The datasets generated and/or analyzed during the current study will be available through Gene Expression Omnibus (GSE 205,415) upon publication.
