# Supplementary material for: Identification of a novel ER-NFĸB-driven stem-like cell population associated with relapse of ER+ breast tumors
Source: Breast Cancer Res. 2022 Dec 8;24:88. doi: 10.1186/s13058-022-01585-1 (PMC9733334; doi:10.1186/s13058-022-01585-1)
Supplement: Supplementary file 1 — Additional file 1: Supplementary Figures. [file 13058_2022_1585_MOESM1_ESM.docx]

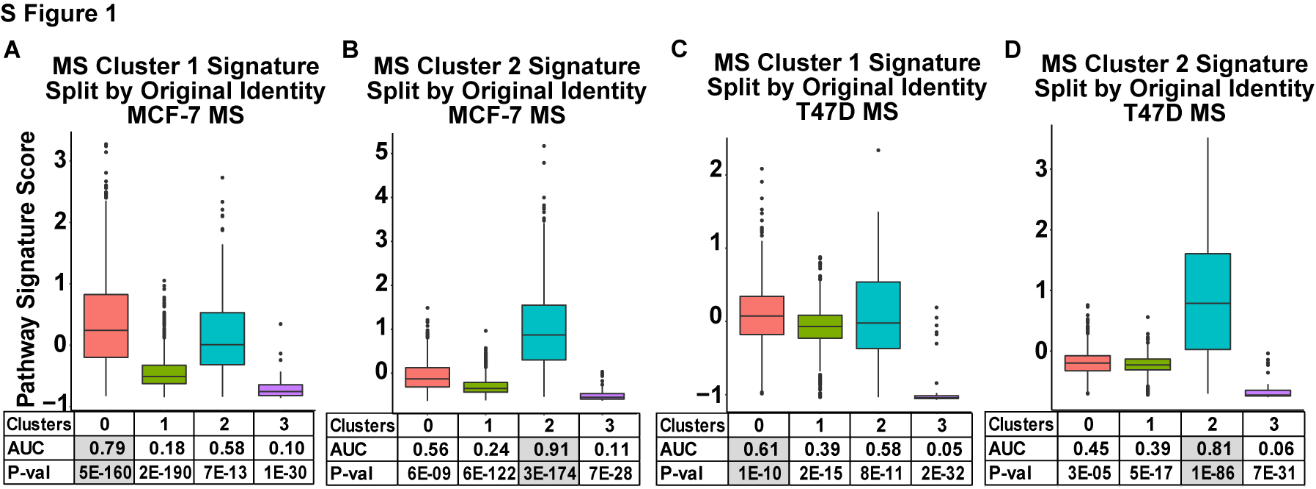


**Supplemental Figure 1.** The MS Cluster 1 Signature and the MS Cluster 2 Signature enrichment in the integrated MCF-7/T47D MS dataset **(Fig. 1E)** split by MCF-7 MS **(A,B)** and T47D MS **(C,D)**. Box plots show signatures’ scores per integrated cluster with significant AUC and P-values indicated in grey.


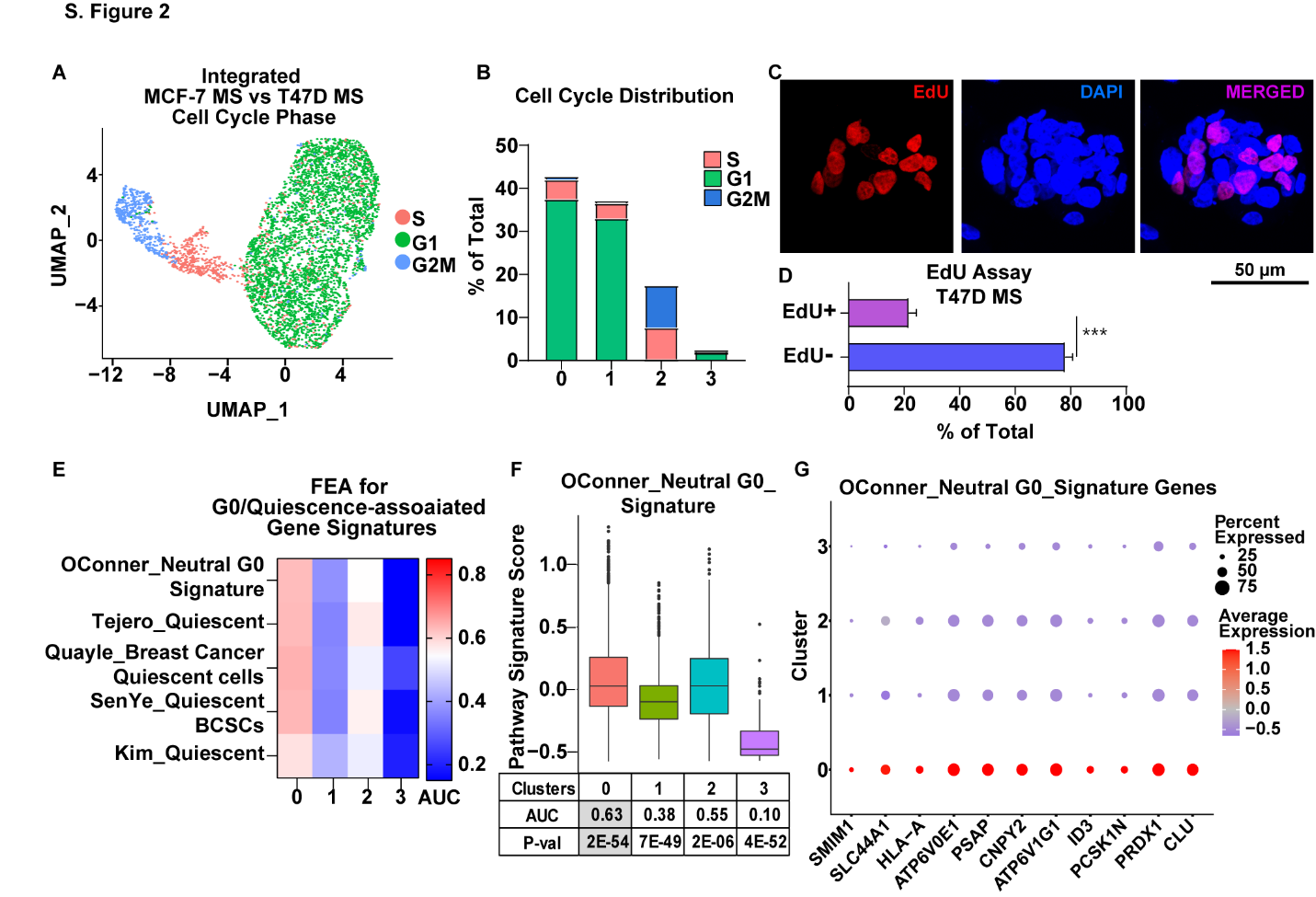


**Supplemental Figure 2. Cell cycle distribution in the integrated MCF-7 T47D MS dataset.**

**(A-B)** Cell cycle analysis was performed on the integrated MCF-7 and T47D MS dataset as described for Fig. 2. Cell Cycle Scoring and Regression vignette provided by Seurat package was used to identify the cell cycle phase at a single cell resolution. **(A)** Bi-dimensional representations of 5739 single-cell transcriptomes colored by cell cycle phase (UMAP) **(A)** with bar graph of cell cycle distribution for each cluster as percent of total number of cells **(B)** are shown. **(C)** An EdU assay was performed on MCF-7 MS. Representative pictures of MS stained for EdU (red) and DAPI (blue) are shown (bar = 50µm). **(D)** A bar chart of EdU+ and EdU- cells presented as a percent of total number of cells from 11 MS is shown. **(E)** FEA was performed on the integrated MCF-7/T47D MS dataset with G0/Quiescence-associated gene signatures with one representative example shown in box plots **(F)**. A detailed description of signatures presented in S. Table 6.1. AUC values are shown in a heatmap, and P-values are presented in Supplemental Table 6.2. **(G)** An expression of genes associated with G0/Quiescence are represented in dot plots, with color representing expression level and size representing the percentage of cells in the cluster expressing the gene.


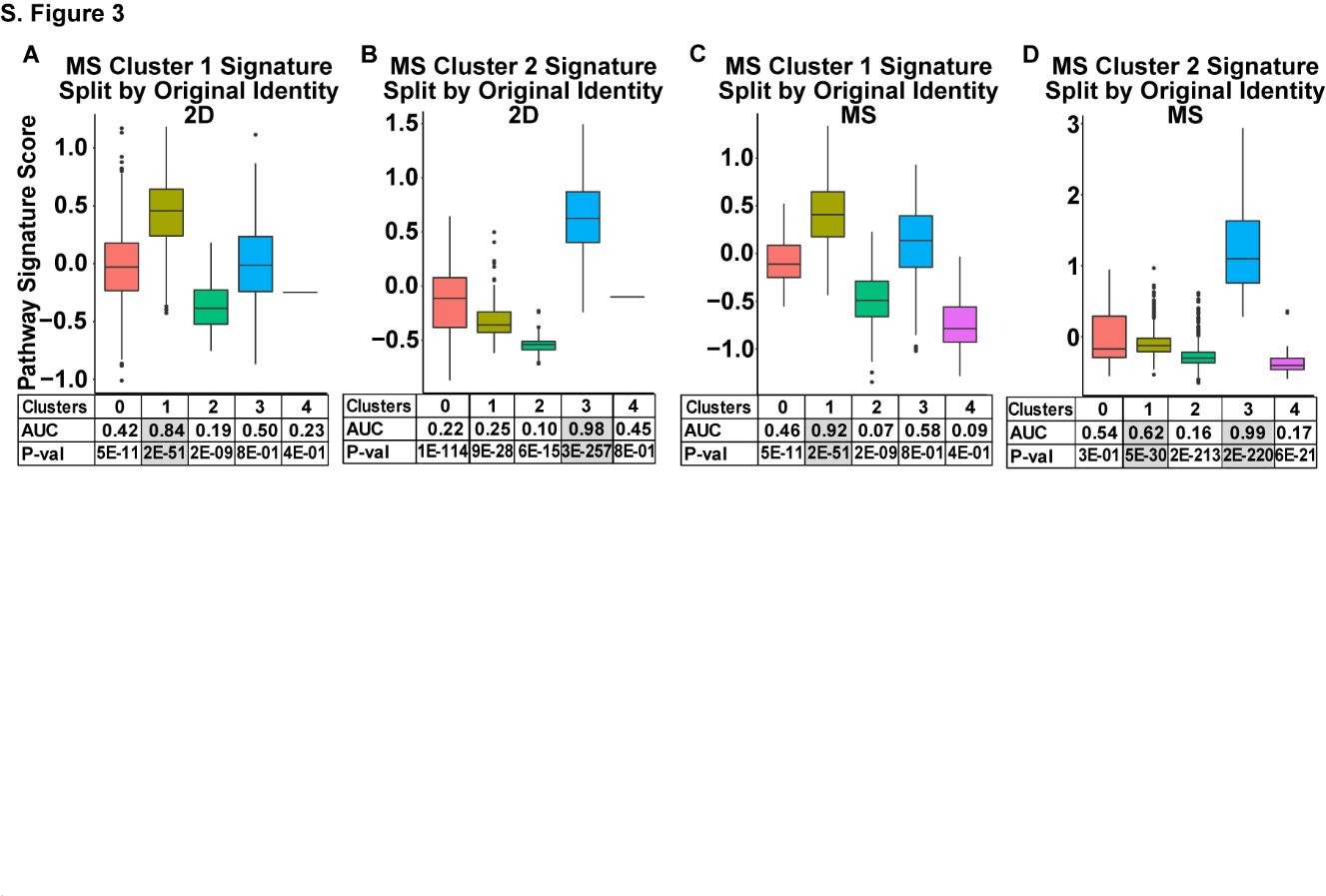


**Supplemental Figure 3**. The MS Cluster 1 Signature and the MS Cluster 2 Signature enrichment in the integrated MCF-7 MS/2D dataset **(Fig. 3A)** split by 2D **(A,B)** and MS **(C,D)**. Box plots show signatures’ scores per integrated cluster with significant AUC and P-values indicated in grey.


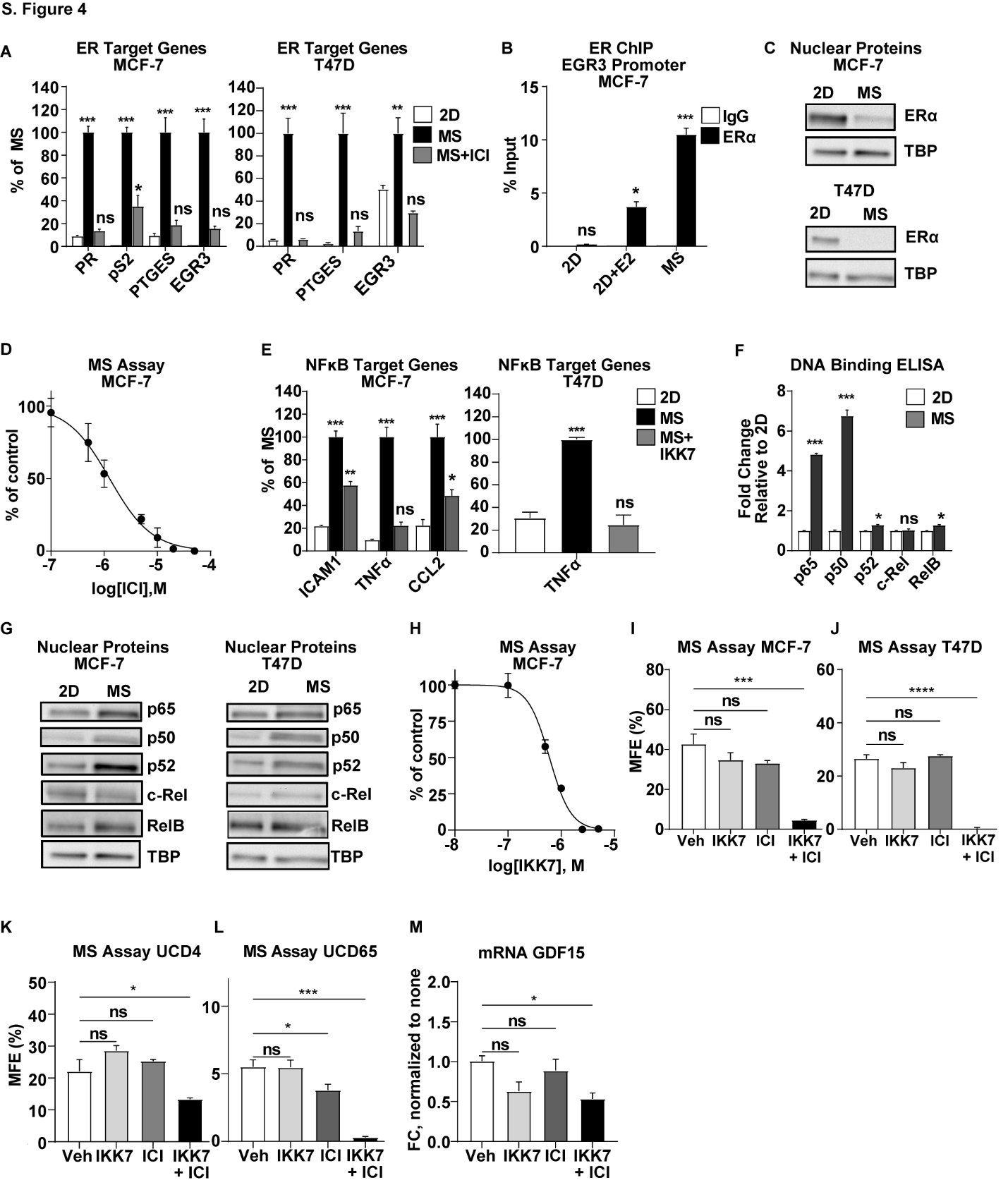


**Supplemental Figure 4. ER and NFĸB are active and required for MS formation.** **(A)** Expression of ER target genes in MCF-7 and T47D cells grown in 2D conditions, in MS conditions, or in MS conditions treated with ICI (1 μM) for the last 24h of the assay. **(B)** ER occupancy at the known ER binding site of early growth response protein 3 (EGR3) in 2D conditions, 2D conditions with E2 treatment, or in MS conditions was assessed by ChIP for ERα or IgG (negative control) followed by QPCR. **(C)** Nuclear localization of ER in MS vs. 2D was detected by Western Blot in MCF-7 and T47D cell lines. TBP served as a loading control. **(D)** MS assay was performed for MCF-7 cells treated with increasing doses of ICI. Log dose response shows MFE for each ICI dose as a percent of control. **(E)** Expression of NFĸB target genes in MCF-7 and T47D cells grown in 2D conditions, in MS conditions, or in MS conditions treated with IKK7 (1 μM) for the last 24h of the assay. **(F)** NFĸB DNA binding activity in MS and 2D nuclear extracts was determined by DNA binding ELISA-based method according to manufacture protocol. **(G)** Nuclear localization of NFĸB family members in MS vs. 2D was detected by Western Blot in MCF-7 and T47D cell lines. **(H)** MS assay for MCF-7 cells treated with increasing doses of IKK7. Log dose response shows MFE for each inhibitor and dose as a percent of control. **(I-L)** MFE was determined for four ER+ breast cancer cell lines treated with sublethal doses of ICI (0.5 μM), IKK7 (0.5 μM) or both. (M) GDF15 expression was measured by QPCR in MCF-7 MS treated with ICI (1 μM) and IKK7 (1μM) for the last 24 hours of the assay. *P<0.05,**P<0.01, ***P<0.001, ns=not significant.


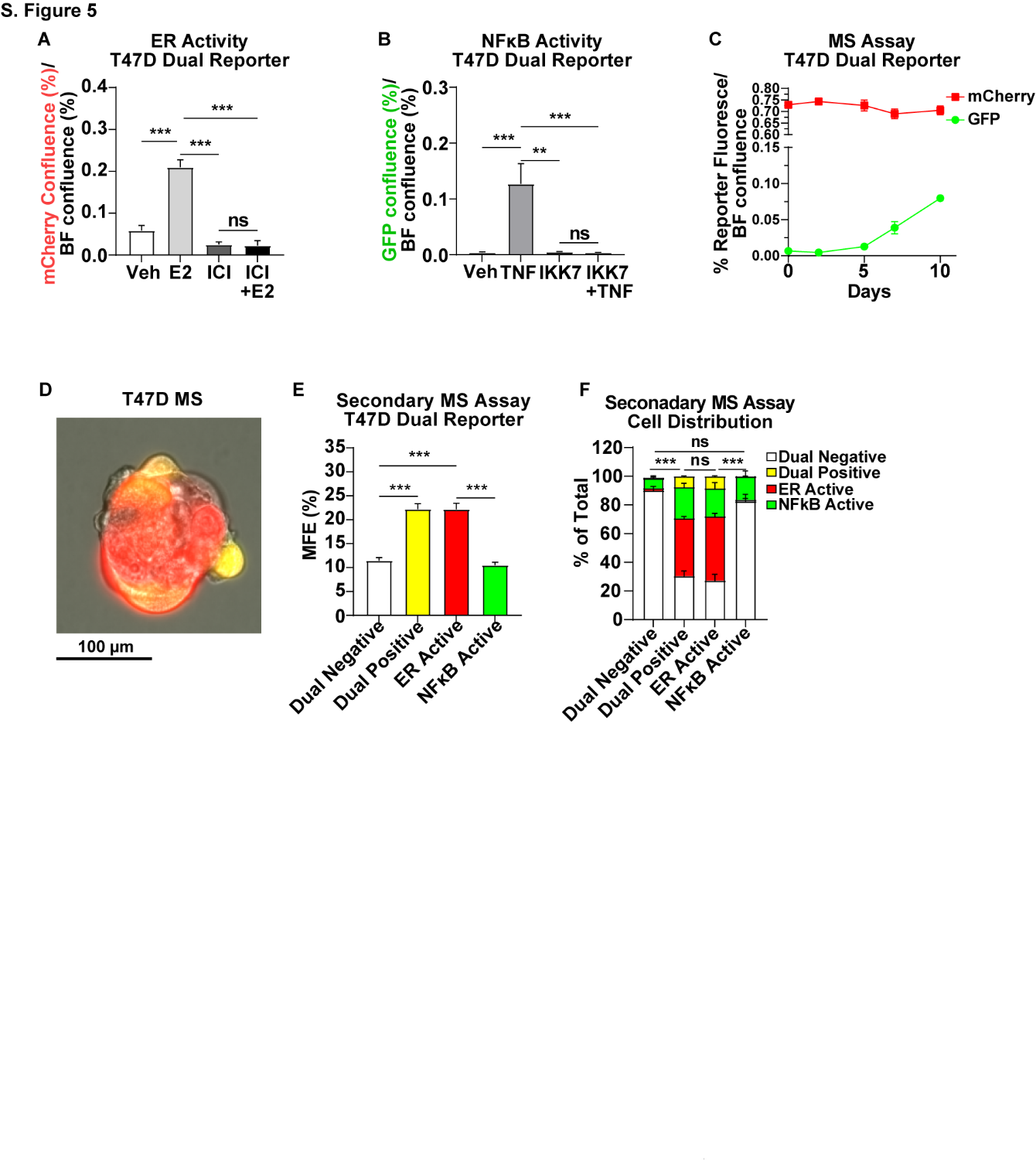


**Supplemental Figure 5. T47D dual reporter cell line to study ER/NFĸB activity in the stemlike population in the MS Cluster 1. (A-B)** ERE-mCherry and NFĸB-RE-eGFP activity was measured in 2D cultured T47D dual reporter cells treated with E2 (10 nM), hTNFα (10 ng/ml), ICI (1 μM), and/or IKK7 (1 μM) for 24h using a Celigo imaging cytometer. Bar charts represent the percentage of mCherry confluence **(A)** and eGFP **(B)** confluence per each treatment group relative to brightfield confluence. **(C)** ERE-mCherry and NFĸB-RE-eGFP activity in T47D MS was measured over time. **(D)** Representative image of MS derived from T47D dual reporter cell line is shown (bar = 100μm). **(E)** Secondary MFE was determined for 4 sorted cell populations derived from primary MS. **(F)** Cell distribution of secondary MS is plotted for each group based on ER-NFkB activity. **P<0.005, ***P<0.001, ns=not significant.

**
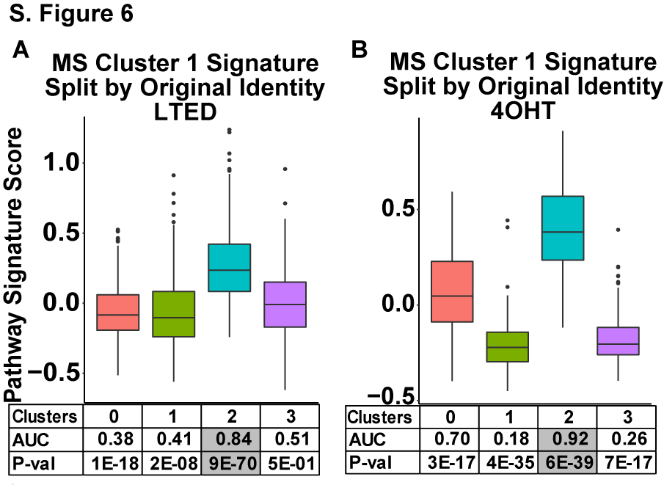
**

**Supplemental Figure 6.** The MS Cluster 1 Signature enrichment in the integrated MCF-7 LTED/4OHT dataset **(Fig. 6A)** split by LTED **(A)** and 4OHT **(B)**. Box plots show signatures’ scores per integrated cluster with significant AUC and P-values indicated in grey.
